# Malaria Vaccines: Recent Advances and New Horizons

**DOI:** 10.1016/j.chom.2018.06.008

**Published:** 2018-07-11

**Authors:** Simon J. Draper, Brandon K. Sack, C. Richter King, Carolyn M. Nielsen, Julian C. Rayner, Matthew K. Higgins, Carole A. Long, Robert A. Seder

**Affiliations:** 1 The Jenner Institute, University of Oxford, Old Road Campus Research Building, Oxford, OX3 7DQ, UK; 2 Center for Infectious Disease Research, 307 Westlake Ave N., Seattle, WA 98109, USA; 3 PATH’s Malaria Vaccine Initiative (MVI), 455 Massachusetts Avenue NW, Suite 1000, Washington, DC 20001-2621, USA; 4 Malaria Programme, Wellcome Sanger Institute, Cambridge, CB10 1SA, UK; 5 Department of Biochemistry, University of Oxford, South Parks Road, Oxford, OX1 3QU, UK; 6 Laboratory of Malaria and Vector Research, NIAID/NIH, Rockville, MD 20852, USA; 7 Vaccine Research Center, NIAID/NIH, Bethesda, MD 20892, USA

## Abstract

The development of highly effective and durable vaccines against the human malaria parasites *Plasmodium falciparum* and *P. vivax* remains a key priority. Decades of endeavor have taught that achieving this goal will be challenging; however, recent innovation in malaria vaccine research and a diverse pipeline of novel vaccine candidates for clinical assessment provides optimism. With first-generation pre-erythrocytic vaccines aiming for licensure in the coming years, it is important to reflect on how next-generation approaches can improve on their success. Here we review the latest vaccine approaches that seek to prevent malaria infection, disease, and transmission and highlight some of the major underlying immunological and molecular mechanisms of protection. The synthesis of rational antigen selection, immunogen design, and immunization strategies to induce quantitatively and qualitatively improved immune effector mechanisms offers promise for achieving sustained high-level protection.

## Teaser

Development of highly effective vaccines against human malaria remains a public health priority. In this Review, Draper et al. describe the latest vaccine approaches to prevent malaria alongside immunological and molecular mechanisms of protection. Rational antigen selection, immunogen design, and novel immunization strategies offer promise for achieving high-level protection.

## Main Text

### Introduction

**Figure 1:**
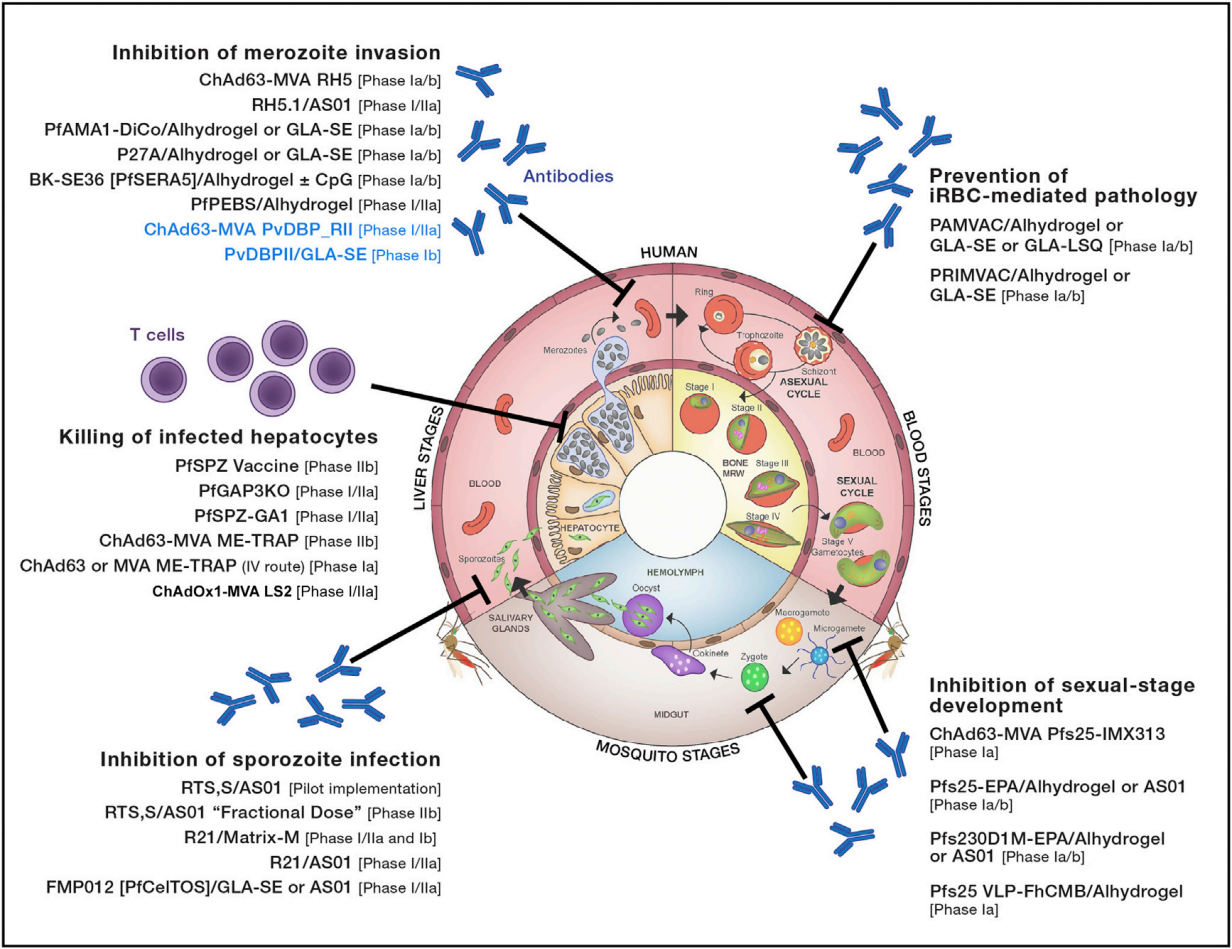
Malaria Vaccine Candidates in Clinical Development Data sources for this figure included the WHO Malaria Vaccine Rainbow Table and Clinicaltrials.gov. Vaccines for *P. vivax* are colored blue. The life cycle figure was adapted from Nilsson et al. (2015).

Modern malaria vaccine development began with seminal studies in mice using irradiated sporozoites (Nussenzweig et al., 1967). Although there is still no licensed product over 50 years later, it is important to remember the scale of the scientific and technical challenges facing those who develop vaccines against such a complex eukaryotic parasite. Moreover, steady progress is being made, especially with regard to breakthroughs in our understanding of the cellular and molecular mechanisms mediating protection in animal models and humans. The revised Malaria Vaccine Technology Roadmap to 2030 (Moorthy et al., 2013) now calls for a next-generation vaccine to achieve 75% efficacy over 2 years against *P. falciparum* and/or *P. vivax* (in an era of renewed global interest toward malaria elimination and eradication), while also retaining its original 2015 “landmark” goal of a first-generation vaccine with protective efficacy of >50% lasting more than 1 year. Achieving this next-generation vaccine goal will necessitate building on the success of current pre-erythrocytic subunit and whole sporozoite-based vaccines, as well as new strategies to add blood-stage or transmission-blocking immunity. Here we review the progress and prospects for a diverse range of approaches targeting different stages of the *P. falciparum* parasite’s complex life cycle (Figure 1), before discussing those in development for *P. vivax*.

### Sporozoite Subunit Vaccines

The most extensively tested vaccine candidate for prevention of *P. falciparum* malaria is RTS,S/AS01; this vaccine directs immune responses against the major circumsporozoite protein (PfCSP) covering the surface of the infecting sporozoite. To accomplish this, RTS,S was designed as a virus-like particle (VLP) comprised of two components: 18 copies of the central repeat and the C-terminal domain of PfCSP fused to hepatitis B virus surface antigen (HBsAg) with extra HBsAg in a 1:4 ratio. RTS,S, formulated with the potent liposomal adjuvant system AS01 from GlaxoSmithKline, is the only vaccine that has demonstrated protective efficacy against clinical malaria in a Phase III clinical trial (Rts, 2015), although protection is partial, wanes over time, and may be age dependent (protection was lower in infants 6–12 weeks of age than in young children 5–17 months old). In the latter, receiving three vaccinations in a 0-1-2 month schedule, the incidence of clinical malaria was reduced by 51% over the first year of follow-up post-dose three [95% CI 48%–55%]. Over 48 months of follow-up, efficacy was 26% [95% CI 21%–31%], and among children receiving a fourth dose at month 20 (18 months post-dose three), efficacy was 39% [95% CI 34%–43%]. A small Phase II study, which followed several hundred children who received the three-dose regimen over 7 years, suggests that there may also be a shifting or rebound in malaria incidence 5 years post-vaccination (Olotu et al., 2016). Results of a larger long-term follow-up study to the Phase III efficacy and safety trial are expected later this year. According to the World Health Organization (WHO), two safety signals (meningitis, cerebral malaria) emerged from the Phase III trial, for which the cause is unknown and they noted a confirmed risk of febrile convulsions within 7 days of vaccination in the 5–17 month age category, all of which resolved without long-term sequelae (WHO, 2016). Following a positive opinion from European regulatory authorities in July 2015, WHO recommended large-scale pilot implementations to further evaluate the feasibility of delivering four doses, the vaccine’s potential for reducing childhood deaths, and to provide additional data on safety in the context of routine use. The pilot implementation program will include robust safety surveillance of these and other safety signals (Klein et al., 2016) that could not be adequately assessed in the Phase III trial due to very low mortality in the trial overall.

**Figure 2:**
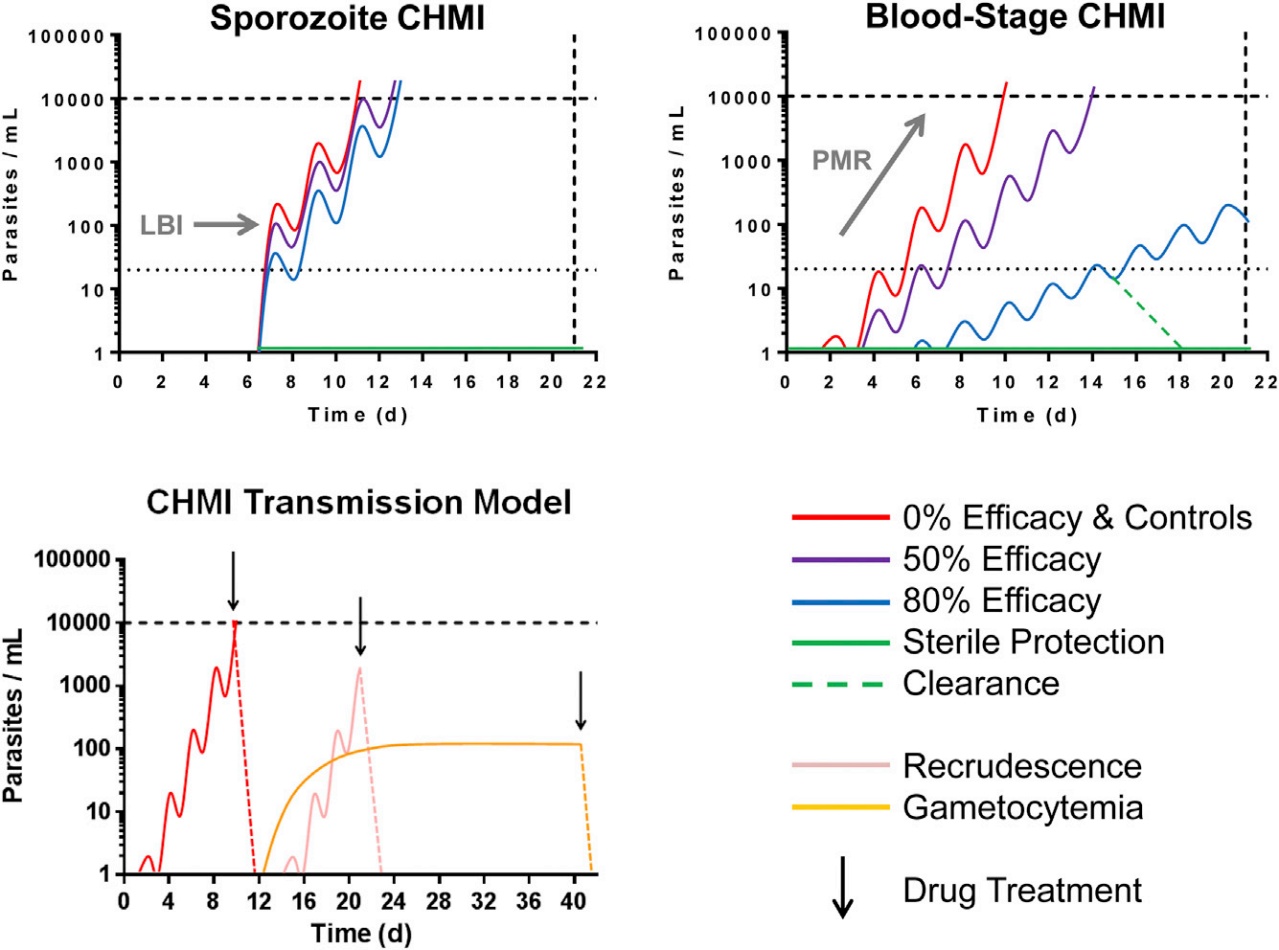
CHMI Models for Vaccine Efficacy Testing Blood-stage parasitemia is monitored by qPCR with lower limit of quantification ∼20 parasites/mL blood (black dotted line), typically for a 21 day study period. Malaria-naïve volunteers are usually diagnosed and treated at ∼10,000 parasites/mL when patent by thick-film microscopy (black dashed line). Following sporozoite CHMI, sterile protection is measured or a partial vaccine effect can be assessed by analysis of the liver-to-blood inoculum (LBI) leading to a delay in time to diagnosis. For blood-stage CHMI, an inoculum of ∼1,000 parasites is administered IV on day 0, with parasites growing ∼10-fold per 48 hr (red line) (Payne et al., 2016). For an effective blood-stage vaccine, a reduction in the parasite multiplication rate (PMR) would be expected. To assess transmission, CHMI is initiated by sporozoites or blood-stage inoculum (the figure depicts the latter). A low-dose drug regimen is used to treat asexual parasitemia, followed by a curative regimen if recrudescence occurs. A wave of gametocytemia then ensues, with all volunteers receiving drug treatment to clear parasites at the end of the study period (Collins et al., 2018).

One of the most important imperatives for future improvements to RTS,S/AS01, and all next-generation malaria vaccines, is to extend the period of protection, which will require further understanding the mechanisms of vaccine-induced efficacy. While a definitive immune mechanism remains to be determined for RTS,S/AS01, the existing data strongly suggest that high antibody concentrations against the NANP amino acid repeats are closely associated with protection, and waning of such responses is likely to be responsible for decreasing efficacy (White et al., 2015). A direct mechanistic link between a monoclonal antibody (mAb) against the NANP epitope (isolated from a subject immunized with RTS,S [Oyen et al., 2017]) and protection will be tested soon following passive transfer and controlled human malaria infection (CHMI) (Figure 2). RTS,S also contains the C-terminal region of PfCSP; however, the role of antibody responses to this region remains unclear as a recent study showed that a number of such mAbs, obtained from a human subject immunized with sporozoites, are not protective in a mouse model (Scally et al., 2018). In addition to antibody, CD4^+^ T cell responses have been suggested to have some role in protection (Kazmin et al., 2017). In some support of this, the C terminus of PfCSP, present in RTS,S, contains two well-defined CD4^+^ T cell epitopes, and a genetic analysis of the Phase III trial indicates a significant sieving effect with modestly lower efficacy for C-terminal sequence unmatched strains (Neafsey et al., 2015). However, a definitive immune mechanism for this effect remains to be determined, and for now the major limitation of RTS,S appears to be maintaining the high antibody levels.

It therefore seems critical for future PfCSP-based subunit vaccines to take into account the parameters of vaccine-induced antibody concentration decay. RTS,S is administered with the adjuvant AS01—the leading formulation for induction of high antibody concentrations in humans. Peak polyclonal anti-NANP serum IgG responses after the final immunization averaged ∼150 μg/mL in malaria-naive adults (Kester et al., 2009), and in the Phase III trial antibody levels declined sharply with initial half-life of ∼40 days followed by a period of slower decline of ∼600 days (White et al., 2015). The requirement for such high and sustained antibody concentrations to mediate durable protection poses a substantial challenge. The impact of novel adjuvants and vaccine delivery platforms may help to provide a solution in the future, especially if they can be demonstrated to skew immune responses toward improved induction of long-lived plasma cells. Antibody decay parameters for an HIV envelope protein delivered with eight different clinical adjuvants have been studied in non-human primates (NHP) (Francica et al., 2017), and the data demonstrate some differences in how adjuvants can influence durability. Nevertheless, improving upon the magnitude of antibody responses by adjuvants other than AS01 may be difficult to achieve. Consequently, an alternative strategy to achieve sustained protection with lower antibody concentrations is by improving the potency or breadth of the polyclonal antibody (pAb) response. The induction of pAb functional at lower concentrations should result in improved vaccine efficacy during a longer segment of the IgG decay curve. Altered vaccine regimens or identification of new neutralizing epitopes on PfCSP could guide such an approach, given that RTS,S does not contain the N-terminal region and portions of the repeat region. Similarly, a more detailed understanding of the contribution made by antibody Fc-mediated effector functions as well as binding parameters, such as affinity, to sporozoite blockade will be important when seeking to design vaccines that induce optimal pAb potency.

Interestingly, the most recent advance in RTS,S/AS01 vaccine development has been made through a modification of dose and schedule: 10/16 (62%) volunteers given the full dose at the standard 0-1-2 month regimen were protected against CHMI 3 weeks after the last immunization; in contrast, 26/30 (86%) volunteers were protected when the third vaccine administration occurred 6 months after the second with the dose reduced to one-fifth of the original dose (“fractional dose,” Fx). Immunological analysis to determine the mechanism for these findings showed that the titers of anti-NANP antibody were similar between the two groups but the Fx regimen may affect antibody avidity, somatic hypermutation, and isotype switching (Regules et al., 2016). While it remains unclear whether the prolonged interval and/or the reduced dose of the final vaccine are mediating these effects, the data raise interesting questions about how this simple alteration controls antibody quality. However, it is important to note that while short-term protection was strikingly improved with the Fx regimen, only 3/7 subjects were protected following secondary CHMI 8 months later. These data suggest that durability of protection will need to be further assessed in future clinical trials and likely improved and, importantly, it remains to be determined whether this altered schedule will lead to improved protection in the field.

Important new information will also emerge from clinical testing of next-generation vaccine designs. The R21 vaccine is composed of a single subunit (equivalent to “RTS” alone without the 4-fold excess HBsAg) (Collins et al., 2017). The display of a higher proportion of PfCSP and less HBsAg per VLP may lead to improved anti-NANP IgG responses in comparison to RTS,S. Clinical testing is underway using Matrix-M or AS01 adjuvants. In an alternative approach, full-length PfCSP containing the N-terminal non-repeat region has been manufactured for clinical testing (Genito et al., 2017), providing the potential to induce antibodies against additional epitopes (not present in RTS,S) that show anti-parasitic function in mice (Espinosa et al., 2015).

**Figure 3:**
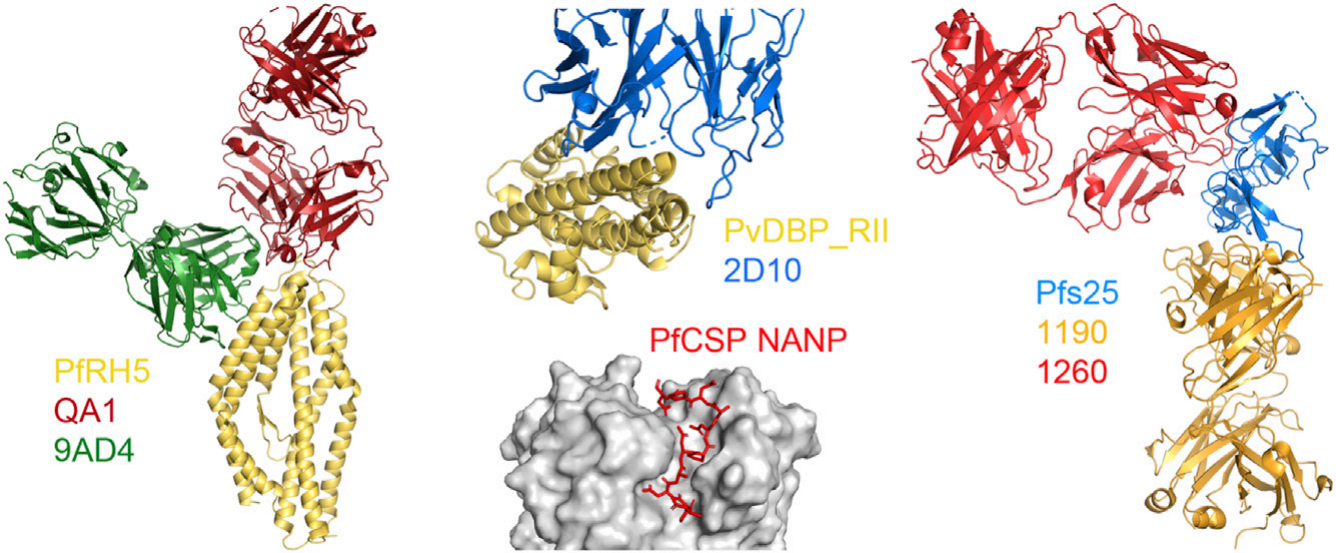
A Structure-Guided Approach to Malaria Vaccine Development Structures of essential *Plasmodium* surface proteins in complex with Fab fragments from mAbs provide a potential starting point for structure-guided vaccine development. Inhibitory mouse mAbs (9AD4 and QA1) binding on PfRH5 are shown (Wright et al., 2014). A mouse binding-inhibitory antibody (2D10), which can prevent PvDBP_RII from binding to human erythrocytes, has been shown to bind to the tip of its third subdomain (Chen et al., 2016). Antibodies that bind the NANP repeats of PfCSP prevent hepatocyte invasion by sporozoites (Oyen et al., 2017). Structural studies have also revealed Pfs25 bound to six different mouse mAbs, identifying two transmission-blocking epitopes, illustrated by the complexes with 1190 (epitope I) and 1260 (epitope II) (Scally et al., 2017).

A major new approach to the field of vaccine research and development for viral proteins such as HIV, influenza, and RSV has been structure-based vaccine design (Kwong, 2017). A starting point is the isolation of a mAb from humans exposed to a given infection or by a vaccine that shows potent neutralizing function. The structure of the epitope bound by the mAb is determined at atomic resolution and this information is used to design immunogens with the goal of eliciting antibodies with functional activity. Structure-guided vaccine design is just beginning for malaria target antigens, including PfCSP (Figure 3). A detailed molecular picture of protein conformations within the full-length PfCSP as well as the binding characteristics of anti-NANP mAbs, derived from RTS,S vaccinees, are providing blueprints for advanced vaccine designs (Oyen et al., 2017). A novel subclass of highly potent anti-PfCSP antibodies, which bind a unique and conserved site of vulnerability found at the junction between the R1 cleavage site and the repeat region, has also been recently reported. These “junctional-binding” mAbs appear to be superior to comparator anti-NANP antibodies for protection *in vivo* as assessed against *P. falciparum* infection in humanized liver-chimeric mice (Kisalu et al., 2018, Tan et al., 2018). Given that RTS,S contains NANP repeats but lacks the full junctional epitope, it will be interesting to see whether these emerging structure-function data can now be harnessed for superior immunogen design leading to more potent anti-PfCSP pAb responses.

Equally important is the identification of non-PfCSP sporozoite vaccine antigens. The only other target to enter recent clinical testing is recombinant PfCelTOS, a micronemal secreted-protein (Draper et al., 2015), but no efficacy against CHMI was observed when formulating in GLA-SE adjuvant, although overall immunogenicity was modest. Preclinically, extensive lists of potential antigens have been reported using genomic, proteomic, and transcriptomic-based approaches (Swearingen et al., 2016) alongside immuno-screening of human samples from naturally exposed or experimentally immunized individuals (Davies et al., 2015). However, prioritization of these targets requires an anti-sporozoite screening assay that closely associates with clinical protection. Defining such an assay remains a critical challenge for the future.

### Whole Sporozoite Vaccines

Various whole sporozoite vaccine (WSV) strategies have demonstrated high levels of protection in preclinical animal models and humans against homologous CHMI, with current studies aiming to define efficacy in malaria-endemic populations, as well as the breadth and duration of protection. WSV rely on administration of live-attenuated sporozoites that cease development at various stages prior to the fulminant blood-stage infection or live sporozoites that reach the blood but are eliminated by drugs. Preclinical studies in rodents show that the timing of arrest has major implications for the breadth and potency of the immune response.

Radiation-attenuated sporozoites (RAS), which were the first WSV studied in both rodents and humans, arrest at random points early in liver-stage development and were first shown to confer protection in humans when administered by mosquito bites. A major advance for vaccine development was the ability to isolate a purified, aseptic, and cryopreserved product of RAS for clinical trials (“PfSPZ Vaccine”). A subsequent scientific advance showed that intravenous (IV) administration of PfSPZ Vaccine was required for inducing potent immunity in humans (Seder et al., 2013) and was associated with robust tissue-resident PfSPZ-specific CD8^+^ T cell responses in the livers of NHP as compared to subcutaneous immunization (Epstein et al., 2011). Indeed, subcutaneous administration showed limited protection against CHMI. These studies provided the first evidence for using IV administration of a preventive vaccine in humans. More recent results with PfSPZ Vaccine from sub-Saharan Africa have been mixed but encouraging. A trial in Malian adults reported that PfSPZ Vaccine was well-tolerated, safe, and easy to administer by direct venous inoculation (DVI) of healthy adults in the clinical trial setting. The proportion of participants with any infection from 28 days after the fifth vaccination to the end of the malaria season (20 weeks) was lower in the vaccinated group than in the control group (HR 0.71, 95% CI 0.53–0.92) (Sissoko et al., 2017). These data suggest that WSV can provide some protection against malaria infection during intense transmission (93% infection rate among placebos). However, a direct comparison of the efficacy of this vaccine to other vaccines is limited by differences in trial designs, efficacy endpoints, and statistical approaches. Efficacy in Mali was also lower than following CHMI of U.S. adults with either homologous or heterologous parasites (Ishizuka et al., 2016, Lyke et al., 2017), although better than that seen with heterologous (7G8 strain) CHMI at 24 weeks (Epstein et al., 2017). Notably, both the trial in Mali and another recent trial in Equatorial Guinea (Olotu et al., 2018) reported lower anti-PfCSP IgG responses in contrast to the same vaccine regimen in U.S. adults, which may reflect hypo-responsiveness to vaccination in malaria-exposed adults; on-going studies will seek to overcome this obstacle through dose and regimen optimization. Moreover, further assessment of protection in young infants in Africa is also required, which will also provide more data on the deployability and feasibility of a vaccine requiring DVI administration.

In contrast to RAS, targeted gene deletion of sporozoites provides a means of homogeneous attenuation. The first clinical trial using a *P. falciparum* genetically attenuated parasite (GAP), lacking two genes p52^−^/p36^−^, led to a breakthrough infection (Spring et al., 2013). However, more recent data showed that a GAP vaccine lacking three genes (p52^−^/p36^−^/sap1^−^; “PfGAP3KO”) arrests early in liver-stage development and was safe, fully attenuated, and immunogenic following mosquito-bite delivery in U.S. adults (Kublin et al., 2017). CHMI efficacy data for this and another GAP called PfSPZ-GA1 (lacking two genes b9^−^/slarp^−^) are awaited. Potentially more promising are the next generation of GAP vaccines. Such vaccines would arrest later in the liver, which in rodent models engenders more antigenically diverse immune responses (breadth of immunity) that are efficacious at lower doses and protect against the blood-stage and multiple rodent parasite species (Sack et al., 2015). Making such late-arresting GAPs in *P. falciparum* has proved challenging, but a *P. falciparum* version of a new rodent late-arresting GAP (lacking the genes *plasmei2* and *lisp2*) (Vaughan et al., 2018) is under development.

A final WSV strategy delivers wild-type sporozoites under chloroquine drug cover, thus allowing liver-stage parasites to develop fully prior to being killed upon entry into the blood to prevent clinical illness. This approach, initially called “chloroquine prophylaxis with sporozoites” (CPS), was protective in 100% of volunteers at 8 weeks after final immunization, with 4/6 volunteers still protected following CHMI ∼2 years later (Roestenberg et al., 2011). However, this approach has recently shown limited protection against heterologous CHMI with two different parasite strains (Walk et al., 2017). The mechanistic reason for this is unclear but may relate to strain-specific immunity of the vaccine or inter-strain differences in liver infectivity that affect challenge potency in CHMI compared to conventional homologous CHMI with the NF54 strain. Use of a specific regimen with non-irradiated, cryopreserved sporozoites delivered by DVI under chloroquine cover (PfSPZ-CVac) has recently shown 100% efficacy (in 9/9 volunteers) against homologous CHMI (Mordmüller et al., 2017), and notably the number of sporozoites required for immunization is 10- to 100-fold less than using irradiated PfSPZ. Assessment of this regimen against heterologous CHMI or natural exposure in field studies is underway. A clear challenge with this approach will be overcoming the potential safety issues related to using a live vaccine that requires concomitant drug delivery to prevent disease; nevertheless, CPS remains an extremely powerful approach to study highly effective malaria immunity.

A major effort in all the aforementioned vaccine studies has been to analyze innate and adaptive immunity to determine correlates and mechanisms of protection. In this regard, our understanding of the roles of both antibodies and T cells in mediating WSV-induced immunity has expanded rapidly in recent years. In terms of IgG antibodies, since WSV vaccines contain the natural sporozoite, they can induce a potentially broad range of antigenic specificities including those against PfCSP in its native confirmation, although titers against the latter remain hard to interpret without direct comparison to PfCSP-based subunit vaccines. Nevertheless, serum from PfSPZ Vaccine-immunized volunteers demonstrates functional *in vitro*, complement-fixing anti-sporozoite IgM (Zenklusen et al., 2018), while passive transfer of purified IgG from malaria-naive US subjects that received the PfSPZ Vaccine and were protected can reduce liver infection >90% in humanized liver-chimeric mouse models (Ishizuka et al., 2016). Of note, the *in vivo* functional inhibition was highest from serum obtained immediately after the final immunization and waned over one year. These data suggest that if antibodies do play a role with WSV it would be soon after immunization, and likely act in concert with T cells (as discussed below). Moreover, these studies highlight that WSV provide a platform for discovery of novel antigens and effector mechanisms, and determining whether these responses are dominated by IgG against PfCSP or alternative targets will be of high future priority.

While recent focus has been on antibody responses, extensive evidence reported in rodent models as well as NHP (Doll and Harty, 2014) has shown cytotoxic CD8^+^ T cells as the major effector mechanism responsible for sterile protection after WSV immunization. Killing of an infected hepatocyte relies on antigen-specific CD8^+^ T cells finding the handful of infected hepatocytes—a situation complicated by the unique immunological and architectural environment of the liver as well as the sheer number of hepatocytes to patrol (>10^11^ hepatocytes/human liver). Indeed, rodent studies have demonstrated that large numbers of specialized liver-resident memory T cells (T_RM_) are required to mediate killing of infected hepatocytes. These cells rely on CXCR6 and CD69 to maintain long-term positioning in the liver where they can patrol the sinusoids and respond rapidly to infection (Fernandez-Ruiz et al., 2016, Tse et al., 2014). Emerging data also suggest an important role for γδ T cells in the induction of such responses at the time of WSV immunization (Ishizuka et al., 2016, Zaidi et al., 2017). Unfortunately, although accessible in NHPs, liver-resident cells are missed with sampling of peripheral blood, likely explaining the modest efficacy of peripherally administered WSV as well as why a clear peripheral CD8^+^ T cell correlate of protection in WSV clinical trials has been elusive.

### Liver-Stage Subunit Vaccines

Generating strong CD8^+^ T cell responses against parasitized hepatocytes with a subunit vaccine requires quite different immunization platforms. To-date, recombinant replication-deficient viral vectored vaccines, especially a chimpanzee adenovirus serotype 63 (ChAd63) prime followed by a boost with modified vaccinia virus Ankara (MVA), have emerged as the most advanced strategy reaching Phase IIb field trials (Ewer et al., 2015). The most widely tested vaccine insert in ChAd63-MVA comprises the thrombospondin-related adhesion protein linked to a multi-epitope string (ME-TRAP). Vaccination provides ∼20%–25% sterile protection against CHMI that associates with a peripheral CD8^+^ T cell response (Ewer et al., 2013). The same vaccine reduced the risk of infection in Kenyan adults by 67% (95% CI 33%–83%) over a short follow-up period (Ogwang et al., 2015), but no efficacy was observed in Senegal (Mensah et al., 2016). Efficacy assessment of this vaccine in the 5- to 17-month-old target age group remains on-going, with trials reporting more promising immunogenicity in contrast to African adults (Bliss et al., 2017).

It also remains unclear whether ME-TRAP is the best choice of antigen. CHMI studies with ChAd63-MVA vectors encoding other well-characterized antigens (PfCSP, and the blood-stage antigens PfMSP1 or PfAMA1) failed to improve on ME-TRAP (Hodgson et al., 2015, Sheehy et al., 2012), while a DNA prime–human adenovirus serotype 5 (AdHu5) boost regimen using PfCSP and PfAMA1 achieved a comparable level of sterile protection in 4/15 (27%) volunteers (Chuang et al., 2013). Ideally, next-generation strategies would therefore seek to identify antigens that provide better MHC class I presentation on the infected hepatocyte surface, as this is required for CD8^+^ T cell recognition and killing of infected hepatocytes (Huang et al., 2015). Studies using WSV have suggested that sporozoite surface proteins as well as liver-stage exported proteins can be more efficiently presented and targeted than parasite-cytoplasmic proteins (Doll et al., 2016, Montagna et al., 2014). However, the potential list of antigens remains limited by an incomplete knowledge of the proteins expressed during the liver-stage of *P. falciparum*. The major limitation for both transcriptomic profiling and identification by mass spectrometry of peptide epitopes eluted from MHC molecules is a suitable and sufficient source of infected hepatocytes. New developments such as humanized liver-chimeric mouse models, *in vitro* liver-stage culture methods, and sorting infected hepatocytes labeled with brightly fluorescent *P. falciparum* lines will undoubtedly expand the repertoire of liver-stage candidate antigens in the coming years (Longley et al., 2015a). In the meantime, on-going efforts continue to screen peripheral T cell responses for antigen reactivity from immune volunteers immunized by WSV strategies, although these are often hampered by lack of statistical power and suitable reagents with which to measure weak responses to many hundreds of potential antigens. Moreover, once identified, preclinical assays to prioritize antigens are challenging given that *P. falciparum* does not infect small animals, and there is no robust *in vitro* assay to measure T cell-mediated killing of liver-stage parasites. A recent alternative to emerge has seen the use of *P. berghei* rodent malaria parasites transgenic for *P. falciparum* liver-stage antigens of interest. Although an imperfect model, such studies have identified antigens that appear to afford greater CD8^+^ T cell-mediated protection than PfTRAP following immunization—including PfLSA1 and PfLSAP2 (Longley et al., 2015b). A Phase I/IIa clinical trial of ChAdOx1-MVA vectors encoding both these antigens in an insert called LS2 is on-going.

Aside from antigen selection, recent evidence also suggests that secondary antigen exposure in the tissue of interest is critical for engendering a T_RM_ cell population. Thus, for liver-stage subunit vaccines, a path forward may be so-called “prime-target” strategies. In this scenario, T cell responses are primed by peripheral vaccination, and then targeted by a boost with a liver-directed vector, such as IV-delivered viral vector, which serves to “target” and educate the T cells to remain in the liver. These strategies have so far shown promise in mouse models of malaria (Fernandez-Ruiz et al., 2016), with results of the first Phase Ia trial to assess IV-delivered ChAd63 and MVA ME-TRAP awaited.

### Blood-Stage Vaccines

Naturally acquired immunity (NAI) to malaria arises through repeated exposure to blood-stage parasite diversity, generation of a broad antibody repertoire against merozoites and infected erythrocytes, and a complex interplay of inflammatory and immuno-regulatory cellular responses. A chemically attenuated whole-parasite blood-stage vaccine (Raja et al., 2017) and subunits against pregnancy-associated malaria using VAR2CSA (Pehrson et al., 2017) have been recently developed that may replicate aspects of NAI, with results awaited. Otherwise, extensive efforts over recent decades have focused on a small handful of well-studied merozoite antigens (usually dominant targets of NAI), seeking to induce antibodies that block erythrocyte invasion; however, these have all struggled to achieve convincing efficacy in clinical trials. Some have also challenged the merits of this vaccine approach given that naturally immune individuals with asymptomatic parasitemia contribute to malaria transmission. In this regard it is particularly encouragingly for the field that new approaches are now emerging that target a small number of highly conserved merozoite antigens that do not appear subject to significant natural immune evasion, challenging the paradigm that blood-stage vaccine strategies should aim to mimic NAI. The bar for such vaccines is high, necessitating much stronger blockade of merozoite invasion than routinely achieved by natural exposure. However, this “non-natural” form of malaria immunity could curtail blood-stage parasite carriage, prevent disease, and development of gametocytes, thereby aligning the goals to prevent both malaria disease and transmission.

Given that this field has struggled, it is important to consider the challenges that have faced vaccine candidates targeting immuno-dominant merozoite antigens. Most notably, these have suffered from the induction of strain-specific pAb responses, which cannot cover substantial levels of antigenic polymorphism and/or redundant invasion pathways. Moreover, the kinetics of merozoite invasion have important implications for all candidate vaccines, and these have perhaps been under-appreciated in the past. *In vitro* studies show that merozoites invade erythrocytes in <1 min (Weiss et al., 2016), with some antigens released from organelles and only exposed for a fraction of this time. These events are so rapid that extremely high concentrations of antibody are required to overcome this constraint imposed on antibody-antigen binding. Concentration (quantity) as well as functional activity or “quality” (driven by target antigen biology, epitopes recognized, antibody affinity, avidity, and subclass) will together determine the efficacy of vaccine-induced pAb. Substantial improvements in both parameters are now required for next-generation vaccines, if they are to achieve high-level control of merozoite invasion and clearance in humans (i.e., reduce multiplication per cycle to <1). On-going efforts to address these challenges with the well-studied polymorphic merozoite antigen PfAMA1 have focused on cocktails of alleles, seeking antibodies against conserved epitopes and hence breadth of parasite coverage (Sirima et al., 2017). However, given that inhibition of homologous parasites is not enhanced, an improved “quality” of the pAb is still required. In this regard, one promising development has used vaccination with PfAMA1 in complex with its peptide ligand PfRON2, leading to improved *in vivo* protection in *Aotus* monkeys (Srinivasan et al., 2017); consistent with a qualitative shift in the pAb related to epitopes recognized around the PfRON2 binding site.

More encouragement for this field, as discussed above, has come from the recent identification of merozoite antigens that appear to overcome the long-standing difficulties of polymorphism and redundancy—the most advanced of which is the *P. falciparum* reticulocyte-binding protein homolog 5 (PfRH5), which forms an essential interaction with basigin (CD147) on the erythrocyte surface during invasion (Crosnier et al., 2011). The high degree of PfRH5 sequence conservation is associated with low-level natural immune pressure, coupled with functional constraints linked to basigin binding and host erythrocyte tropism (Draper et al., 2015). Vaccination of animals with full-length PfRH5 induces pAb that inhibit *in vitro* all *P. falciparum* lines tested to date, while *Aotus* monkeys are protected by PfRH5 vaccination against a stringent heterologous challenge. In this case, anti-PfRH5 serum IgG antibody concentration and functional activity, measured using purified IgG in the standardized cell-independent *in vitro* assay of growth inhibition activity (GIA), were both associated with protective outcome (Douglas et al., 2015). Two further NHP studies have identified the assay of GIA as an *in vitro* correlate of vaccine-induced *in vivo* protection (Mahdi Abdel Hamid et al., 2011, Singh et al., 2006), with passive transfer of a GIA-positive anti-PfRH5 human mAb also showing *in vivo* protection in a humanized mouse model (Foquet et al., 2018). These data suggest a threshold level of GIA is required to protect and provide a benchmark to aim for in future human clinical studies.

**Figure 4:**
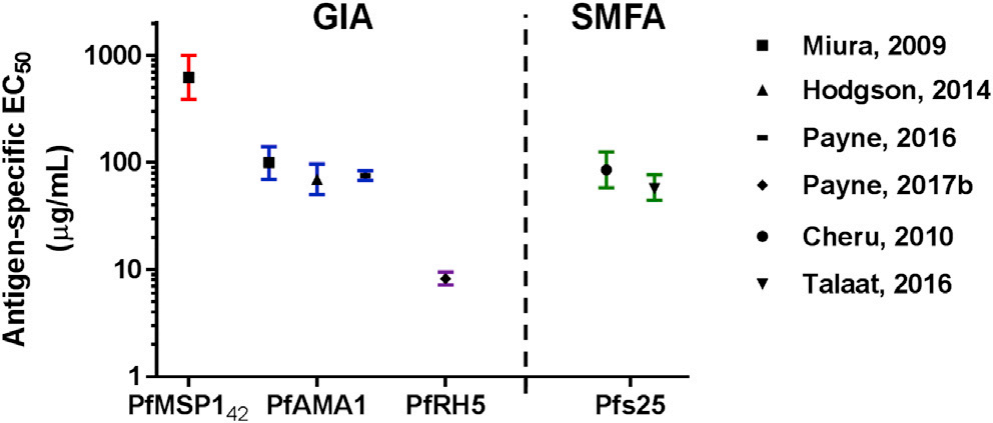
Functional Analysis of Human Antigen-Specific IgG Reported EC_50_ [±95% CI] data from Phase Ia clinical trials of vaccine-induced human antigen-specific IgG, as assessed for functional activity against blood-stage (GIA) or sexual-stage parasites (SMFA). Vaccine-induced polyclonal IgG responses are carefully quantified using affinity-purified ELISA standards (Cheru et al., 2010, Miura et al., 2009) or calibration-free concentration analysis (Hodgson et al., 2014).

Careful quantification of vaccine-induced pAb responses has now allowed for informative comparison between antigens using this functional assay (Figure 4). Anti-PfRH5 antibodies appear to block erythrocyte invasion to high efficiency, requiring lower antigen-specific pAb concentrations to give 50% GIA (EC_50_) than against PfMSP1 and PfAMA1—a consistent observation through animal models and now vaccinated humans (Payne et al., 2017b). Such quantitative analyses offer hope that highly susceptible antigen specificities can now be rationally identified to overcome the challenge of merozoite invasion kinetics. Analysis of anti-PfRH5 mouse mAbs also identified potent neutralizing clones, with the best EC_50_ in the range of 10–15 μg/mL, although room for improvement remains, given that this is 50-fold less potent than anti-basigin mAbs where no kinetic constraint is imposed on antibody binding (Zenonos et al., 2015). These potent anti-PfRH5 clones bind within or extremely close to the basigin binding site (Wright et al., 2014), suggesting future strategies for structure-guided immunogen design (Figure 3).

Furthermore, a multi-protein complex has now been elucidated containing PfRH5 as well as PfRipr and PfCyRPA, both of which are essential, highly conserved molecules that elicit functional antibodies (Chen et al., 2011, Reddy et al., 2015). This soluble PfRH5-PfRipr-PfCyRPA invasion complex is required for triggering Ca^2+^ release and establishing the tight junction (Volz et al., 2016), with the PfP113 surface protein binding the N terminus of PfRH5 to provide an anchor to the merozoite surface (Galaway et al., 2017). Further complexities surrounding PfRH5 biology have also been described, such as interactions between cyclophilin B and basigin as well as PfRhopH3 and PfRH5 (Prakash et al., 2017), or PfRAP2 and basigin (Zhang et al., 2018b). Importantly, targeting these multiple components may improve vaccine potency; indeed antibodies against PfRH5 and PfCyRPA can synergize (Reddy et al., 2015), with recent structural analyses identifying inhibitory epitopes on PfCyRPA (Chen et al., 2017). Alternatively, targeting discrete temporal steps during invasion may provide another route to rational vaccine improvement. Indeed, a merozoite antigen screen by reverse vaccinology identified targets that elicit functional IgG, including PfRAMA whose antibodies synergized with anti-PfRH5 and anti-PfCyRPA antibodies (Bustamante et al., 2017). In parallel, other novel antigen combinations continue to be identified by using protein libraries to screen African sera (Morita et al., 2017), while further candidates are expected to arise from elegant experimental genetic approaches (Zhang et al., 2018a). After many decades of difficulty, the merozoite vaccine field is seeing a resurgence of candidates and approaches, with the first human efficacy data of PfRH5-based vaccines eagerly awaited.

### Transmission-Blocking Vaccines

Unique among malaria vaccine strategies, those targeting the sexual stages do not directly prevent infection or clinical symptoms within the host, but rather impact the parasite’s life cycle in the mosquito vector aiming to prevent sporozoite development and onward transmission. Proof-of-concept was first reported in the 1970s, but substantial progress and political traction has only happened over the last decade, in line with renewed calls for malaria elimination. Notably, while a transmission-blocking vaccine (TBV) would not directly protect an individual, it could have substantial impact: in an endemic population of asymptomatic and/or submicroscopic carriers, a TBV will serve to arrest onward transmission of malaria and thus provide protection to the community as other vaccines do through herd immunity.

The leading TBV antigens include the ookinete surface protein Pfs25, and the gametocyte antigens Pfs48/45 and Pfs230. Antibodies against all three perform well in comparative preclinical studies (Kapulu et al., 2015), with functionality assessed by the standard membrane feeding assay (SMFA) using mosquitoes that are fed cultured gametocytes in the presence of whole serum or purified IgG (notably efficacy of anti-Pfs230 antibodies is also complement dependent). Identification of improved antigen targets (based on proteomic and transcriptomic datasets and protein microarray screens) remains an area of intense research, with studies starting to yield promising results (Stone et al., 2018). Other more recently identified targets of interest include Pfs47, involved in parasite immune evasion in the mosquito vector (Molina-Cruz et al., 2013), and PfHAP2, which is expressed on the male gametocyte and microgamete and is essential for membrane fusion during fertilization (Angrisano et al., 2017).

Pfs25 was the first antigen to progress clinically; however, this Phase Ia trial with Pfs25 protein formulated in Montanide ISA51 led to unacceptable levels of reactogenicity (Wu et al., 2008), meaning subsequent efforts have focused on improved delivery platforms, such as conjugation to exoprotein A of *Pseudomonas aeruginosa* (EPA) (Shimp et al., 2013) or fusion to IMX313, a protein heptamerization technology resulting in nanoparticle formation (Li et al., 2016). A number of candidates have now entered clinical testing (Figure 1), with Pfs25-EPA/Alhydrogel the first to be reported (Talaat et al., 2016). Here, anti-Pfs25 serum antibody levels correlated with activity in the SMFA (with 57 μg/mL [95% CI 45–77 μg/mL] Pfs25-specific human IgG achieving 50% reduction in oocyst intensity), suggesting high-level transmission blocking may require substantial antibody concentrations (Figure 4). Indeed, defining whether a given level of SMFA activity is sufficient to impact malaria transmission in the field remains an intense area of research (Bompard et al., 2017, Miura et al., 2016). Importantly, these studies have been enabled by substantial progress with regard to standardizing the SMFA so that TBV activity can be evaluated by multiple laboratories (Miura et al., 2013). Other labor-intensive studies are now seeking to bridge to other assays used in the field, including the direct membrane feeding assay (DMFA) using gametocytes isolated from naturally infected individuals, direct skin-feeding assays, as well as community-wide clinical endpoints (Brickley et al., 2016). These efforts will lay the foundations for the interpretation of field trial data. Indeed, extension of the Pfs25-EPA vaccine, along with a second EPA-conjugated immunogen composed of the small D1M domain of Pfs230 (MacDonald et al., 2016), to field trials in Mali is now underway using both Alhydrogel and the stronger adjuvant AS01 (Figure 1).

Other preclinical efforts continue to design a Pfs48/45 immunogen that is suitable for clinical biomanufacture with promising results reported for a chimeric antigen: this fuses a “correctly folded” Pfs48/45 region containing the C-terminal 6-cysteine (6C) domain (evaluated by recognition of the highly potent conformation-dependent rat mAb45.1) to the R0 portion of the asexual-stage PfGLURP. The chimera denoted R0.6C is produced in *Lactococcus lactis* and intended for clinical development (Theisen et al., 2017). Other investigations designed to seek the most potent epitopes on TBV targets have recently identified two distinct immunogenic sites in crystal structures of the Pfs25 antigen in complex with antibodies elicited by immunization of human immunoglobulin loci transgenic mice (Scally et al., 2017). These non-overlapping sites can be targeted simultaneously by antibodies to additively increase parasite inhibition, providing a structural blueprint for next-generation immunogen design (Figure 3). In parallel, work is also seeking to array Pfs25 antigen on highly immunogenic VLP scaffolds. A recent study compared chemical cross-linking to the Qβ-bacteriophage, versus, and “plug-and-display” conjugation to the AP205-bacteriophage using the SpyTag/SpyCatcher technology (Leneghan et al., 2017). While the chemically conjugated VLP elicited the highest quantity of antibodies, the plug-and-display VLP elicited the highest quality anti-Pfs25 antibodies measured by SMFA. Extension of this work to two orthogonally reactive split protein pairs is now enabling dual antigen immunization (Pfs25 and Pfs28) on a single synthetic nanoparticle (Brune et al., 2017). These exciting developments in delivery technologies for highly immunogenic arrayed antigens bode well for the future testing of defined malaria antigen combinations and improved immunogens.

### Vaccines for *P. vivax*

While most vaccine development has focused on the major cause of malaria mortality, *P. falciparum*, an effective vaccine could also greatly facilitate elimination of *P. vivax* in many areas of the Americas and Asia-Pacific where standard malaria control tools are facing unique challenges from this parasite’s biology (Mueller et al., 2015). These include dormant hypnozoites in the liver that cause relapsing infections in the absence of transmission; early appearance of gametocytes before onset of clinical symptoms; and a shorter development cycle in the mosquito. As with *P. falciparum* vaccines used for elimination, it is also likely that a *P. vivax* vaccine will be needed for the entire population and not just infants, given that in many settings transmission rates are relatively low and the population largely non-immune.

An effective pre-erythrocytic vaccine could reduce primary infections as well as prevent establishment of hypnozoites, thus reducing the risk of multiple relapses contributing to transmission (White et al., 2017). To-date, two subunit vaccines targeting PvCSP have reached clinical trials, while no WSV formulation has been developed and studies of PvRAS suggest protection is only achieved in subjects who receive very high doses (Arévalo-Herrera et al., 2016). In the case of PvCSP, a soluble protein (VMP001/AS01) was immunogenic in U.S. volunteers but failed to induce sterile protection following *P. vivax* mosquito-bite CHMI; although a significant delay in time to parasitemia was seen in 16/27 vaccinated subjects (Bennett et al., 2016). A VLP similar to RTS,S/AS01, called CSV-S,S/AS01 (Vanloubbeeck et al., 2013), was not progressed; although a particle called Rv21 with a higher density of PvCSP on the VLP surface is progressing to clinical development (Salman et al., 2017). While progress with PvCSP has been slow, exciting technical developments including humanized liver-chimeric mice, *in vitro* culture methods, and the *P. cynomolgi* monkey model are now increasing access to hypnozoites, opening the door for new vaccine candidates targeting these dormant forms (Gural et al., 2018).

At the blood stage, *P. vivax* invasion is restricted to reticulocytes that express the iron importer transferrin receptor 1 (TfR1/CD71) (Malleret et al., 2015) and requires the interaction of the *P. vivax* Duffy-binding protein (PvDBP) with the human Duffy antigen receptor for chemokines (DARC/Fy). Indeed, the only two vivax vaccines in active clinical development (Figure 1) both target the conserved, extracellular, cysteine-rich region II (PvDBP_RII), with the first published data reporting promising immunogenicity in a Phase Ia trial (Payne et al., 2017a); however, detailed interrogation of vaccine-induced antibody responses remains hampered by the lack of long-term *in vitro* culture systems for this parasite. Preclinically, structural studies of PvDBP_RII bound to its receptor and specific mouse mAbs (Chen et al., 2016) are starting to identify important determinants related to receptor-binding blockade and polymorphism-mediated immune escape, thus guiding rational design of next-generation PvDBP_RII immunogens (Chen et al., 2015) (Figure 3). Notably, the PvDBP-DARC paradigm has also been challenged with reports of *P*. *vivax* infection in Duffy-negative individuals (Zimmerman et al., 2013) and a PvDBP gene duplication in parasite isolates (Menard et al., 2013) likely representing a second erythrocyte-binding protein (Hester et al., 2013), although studies have not linked this gene to Duffy-negative infection (Hostetler et al., 2016). Therefore, although the complete molecular basis of *P. vivax* invasion into DARC-negative erythrocytes remains unknown, it may still involve PvDBP. In parallel the complexity of other invasion ligand families, including the reticulocyte-binding proteins (PvRBPs), is being described, with PvRBP2b (a distant homolog of PfRH5—there is no clear *P. vivax* ortholog of this high priority *P. falciparum* target) binding TfR1 as a critical host factor defining *P. vivax* tropism for reticulocytes (Gruszczyk et al., 2018). These new candidates and others from antigen arrays (França et al., 2017), as well as suggestions that cytotoxic CD38^+^ CD8^+^ T cells may target parasites residing within MHC class I-expressing reticulocytes (Burel et al., 2016), provide a growing wealth of targets and strategies on which to build a next-generation blood-stage vaccine.

In the case of transmission blocking, the first TBV to ever enter clinical development targeted *P. vivax* and not *P. falciparum*. This Pvs25H/Alhydrogel protein vaccine elicited antibodies that showed activity in DMFA, but vaccinations in a second trial with Montanide ISA51 adjuvant were halted due to unexpected reactogenicity (Wu et al., 2008). For now, new clinical TBV candidates for *P. vivax* remain awaited.

### Outlook and Anticipated Future Developments

**Figure 5:**
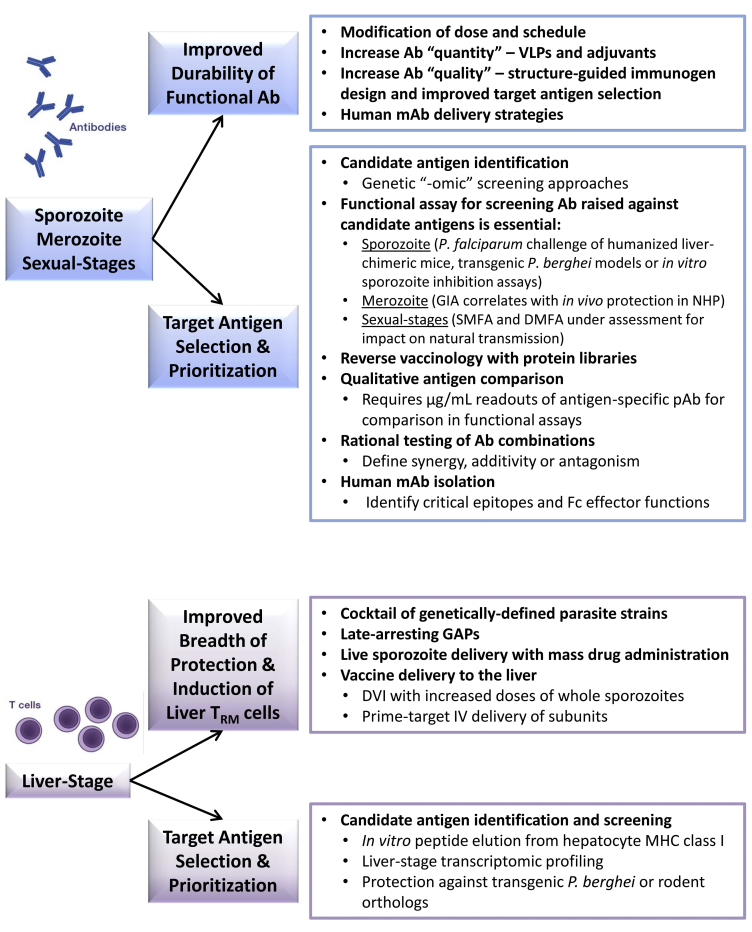
Examples of Approaches to Next-Generation Malaria Vaccine Discovery

Over the next 5 years, it is possible that RTS,S/AS01 will proceed to application for licensure (although review and approval by national regulatory authorities is required for use in the pilot implementation expected to start in late 2018). If successful, this will mark a major milestone in the malaria field. Other vaccines pursuing licensure include PfSPZ Vaccine and R21, although these candidates require additional data. Next-generation vaccines will be essential to follow in these footsteps if higher and sustained levels of protection are to be achieved—either as new or direct improvements to these pre-erythrocytic strategies and/or to provide added blood-stage or transmission-blocking immunity. Achieving this goal will necessitate new strategies, many of which are already in preclinical development and showing promise. These approaches fundamentally differ by their need to induce functional antibodies against sporozoites, merozoites, or sexual stages versus T cell-mediated immunity against the infected hepatocyte; but a blueprint for these activities and key developments likely to be seen in the near future for *P. falciparum* are outlined in Figure 5. It is likely that new clinical vaccine candidates for *P. vivax* will follow based on similar strategies and technologies, but these two major human *Plasmodium* species are only distantly related and have very different biology, so how fruitful this will be waits to be seen.

Alongside efforts to maximize life cycle stage-specific immunity, combining clinical candidates in a multi-stage approach is now an obvious option to address whether additive, or ideally synergistic, protective efficacy can be achieved. Although a recent trial of RTS,S/AS01 used in conjunction with the liver-stage subunit ChAd63-MVA ME-TRAP did not show improved protection against CHMI (Rampling et al., 2016), other preclinical data suggest encouraging synergy when combining pre-erythrocytic and transmission-blocking vaccines (Sherrard-Smith et al., 2018). The increasing number of candidate vaccines acting around the parasite’s life cycle means that these and other multi-stage strategies can now be explored clinically.

Irrespective of life cycle stage, consistent themes and challenges currently pervade the *P. falciparum* malaria vaccine field. For antibody-inducing strategies, these include the need for more durable and/or functional responses to maintain vaccine efficacy as induced IgG responses wane into the memory phase. Responses will likely be improved through rational structure-based immunogen design, optimized immunization regimens, and VLP-based delivery platforms. For T cell-inducing strategies, these need to deliver more durable and effective liver T_RM_ cells. Both approaches need to maintain focus on defining immune mechanism-based correlates of protection to allow identification of the best possible new target antigens and/or combinations, while refining their immunogens to maximize vaccine breadth as well as quantitative and qualitative potency. These considerations are paramount to avoid years of empirical testing and disappointment, and instead, the wealth of recent technological advances now at vaccine developers’ disposal should allow for an informed and integrated approach to next-generation vaccine design. Once coupled with on-going progress in terms of vaccine biomanufacture, human vaccine delivery platforms and efficacy assessment by long-term and heterologous CHMI, the coming years promise to be an exciting time that should engender hope that a highly effective vaccine may be within reach.
